# Long‐term effectiveness and safety outcomes in adults with Fabry disease treated with agalsidase alfa: 20 years of data from the Fabry Outcome Survey

**DOI:** 10.1111/eci.70142

**Published:** 2025-11-06

**Authors:** Derralynn A. Hughes, Guillem Pintos‐Morell, Christoph Kampmann, Christina Anagnostopoulou, Jaco Botha, Siddharth Jain, Kathleen Nicholls, Dau‐Ming Niu, Ricardo Reisin, Michael L. West, Jörn Schenk, Uma Ramaswami, Roberto Giugliani

**Affiliations:** ^1^ Royal Free London NHS Foundation Trust University College London London UK; ^2^ Vall d'Hebron Research Institute Barcelona Spain; ^3^ Medical Consulting Committee, MPS‐Lisosomales Association Barcelona Spain; ^4^ Johannes Gutenberg School of Medicine University of Mainz Mainz Germany; ^5^ Takeda Pharmaceuticals International AG Zurich Switzerland; ^6^ Takeda Development Center Americas, Inc. Cambridge Massachusetts USA; ^7^ The Royal Melbourne Hospital Parkville Victoria Australia; ^8^ University of Melbourne Parkville Victoria Australia; ^9^ Institute of Clinical Medicine National Yang Ming Chiao Tung University Taipei Taiwan; ^10^ Taipei Veterans General Hospital Taipei Taiwan; ^11^ Department of Neurology Hospital Británico Buenos Aires Argentina; ^12^ Department of Medicine Dalhousie University Halifax Nova Scotia Canada; ^13^ Department of Genetics, Federal University of Rio Grande do Sul (UFRGS), Medical Genetics Service, Hospital de Clinicas de Porto Alegre (HCPA), Instituto Nacional de Ciência e Tecnologia em Multiômica Aplicada à Saúde de Precisão (IMASP) Dasa Genomics and Casa dos Raros Porto Alegre Brazil; ^14^ Present address: Medison Pharma Zug Switzerland; ^15^ Present address: KalVista Pharmaceuticals Zug Switzerland

**Keywords:** agalsidase alfa, cardiac, Fabry disease, Fabry Outcome Survey, registry, renal

## Abstract

**Background:**

We present the final report from the Fabry Outcome Survey (FOS) on long‐term effectiveness and safety of agalsidase alfa in adults (≥18 years old).

**Methods:**

FOS was an international, multicentre, observational registry (NCT03289065), designed to enhance the understanding of Fabry disease and improve clinical management. Primary effectiveness endpoints were annualized change in estimated glomerular filtration rate (eGFR) and left ventricular mass index (LVMI), and time to and age at composite morbidity event (comprising renal, cardiac or stroke events) and death. Safety outcomes were also assessed.

**Results:**

FOS included data for 1864 adults (female/male, *n =* 907/957) who received agalsidase alfa only for a median (minimum, maximum) of 6.0 (0, 21.6) years, and 1613 untreated adults (female/male, *n =* 1235/378). At baseline, mean (standard deviation [SD]) eGFR was 94.01 (27.60) mL/min/1.73 m^2^ in treated adults; annualized changes in eGFR (slope [standard error; SE]) remained relatively stable in females and declined slightly in males (−1.07 [.12] vs. −2.17 [.12] mL/min/1.73 m^2^). At baseline, mean (SD) LVMI was 58.25 (25.01) g/m^2.7^ and LVMI (slope [SE]) remained stable (.34 [.16] vs. .38 [.15] g/m^2.7^/year in females and males, respectively). Time (median [95% confidence interval]) from treatment initiation to first composite event was longer for females than males (83.4 [65.7–98.0] vs. 56.3 [45.6–66.7] months); age (median [minimum, maximum]) at death was also higher for treated females than males (69.9 [32.5, 87.7] vs. 59.1 [26.2, 79.6] years). Agalsidase alfa was generally well tolerated.

**Conclusions:**

This report further supports the long‐term effectiveness and safety of agalsidase alfa in adults with Fabry disease.

## Graphical abstract

Long‐term treatment with agalsidase alfa in 1864 adults with Fabry disease in the Fabry Outcome Survey confirmed previously reported beneficial effects on renal function and cardiomyopathy. Over a median (min, max) of 6.0 (0, 21.6) years of treatment, annualized changes in eGFR remained relatively stable in females and declined slightly in males. LVMI remained stable in females and males. Beneficial effects were also seen on morbidity and mortality versus Fabry disease natural history data. Agalsidase alfa was generally well tolerated.

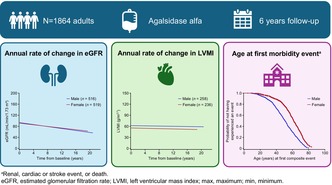

## 1 INTRODUCTION

Fabry disease is a rare X‐linked disease caused by a deficiency in the activity of the lysosomal enzyme α‐galactosidase A, which catabolizes globotriaosylceramide and related glycosphingolipids.^1^ The disease is multisystemic and progressive, with patients developing conditions including angiokeratoma, hypo‐ or hyperhidrosis, neuropathic pain, gastrointestinal symptoms, cornea verticillata, cerebrovascular accidents, cardiomyopathy and renal dysfunction.^1^, ^2^ Life expectancy is reduced in both males and females, with an increased risk of death from renal, cardiovascular or cerebrovascular disease.^3^

The Fabry Outcome Survey (FOS) was an international, multicentre, observational, physician‐directed registry (ClinicalTrials.gov identifier: NCT03289065) sponsored by Shire, a Takeda company, that was initiated in April 2001 to collect data on long‐term clinical outcomes, safety and disease progression in patients receiving agalsidase alfa, as well as disease progression in untreated patients.^4^ FOS was changed to a disease registry including patients receiving agalsidase beta or migalastat in 2016, following a merger with the Fabry International Research Exchange (FIRE).^4^ Previous FOS data publications have demonstrated the long‐term safety and effectiveness of agalsidase alfa in children and adults with Fabry disease.^5^, ^6^, ^7^, ^8^ The FOS database closed on 30 September 2022, and the present analysis represents the final report from FOS on the natural history of Fabry disease and the effectiveness and safety of enzyme replacement therapy with agalsidase alfa only in adult patients.

## 2 METHODS

### 2.1 Study design and patient population

FOS was conducted in compliance with the Declaration of Helsinki, international guidelines for pharmacoepidemiology studies and applicable local regulations, with the approval of the ethics review boards of participating centres and the informed consent of patients and caregivers.

The FOS protocol has been described previously.^9^, ^10^, ^11^ To be eligible for inclusion, patients were required to have a documented diagnosis of Fabry disease. The exclusion criterion was current enrolment in an ongoing blinded clinical trial. The first objective of this present analysis was to evaluate the course of Fabry disease in patients receiving agalsidase alfa only (treated group) or in those who did not receive any Fabry disease‐specific treatment (untreated group). The second objective was to evaluate the safety of agalsidase alfa in patients with Fabry disease.

### 2.2 Assessments

All outcomes were assessed for adults (patients aged ≥18 years), except for eGFR, where the protocol‐specified primary effectiveness endpoint was change in renal function over time assessed by estimated glomerular filtration rate (eGFR) in patients aged ≥16 years. Post hoc renal outcomes were assessed for patients aged ≥18 years for consistency with other endpoints. For renal disease progression, patients were stratified according to baseline eGFR levels (<60 and ≥60 mL/min/1.73 m^2^) and baseline urinary protein levels (<.3, ≥.3 to <1 and ≥1 g/24 h). eGFR was calculated using the Chronic Kidney Disease Epidemiology Collaboration equation for patients aged ≥18 years^12^ and the Counahan–Barratt formula for those aged <18 years.^13^, ^14^ Per protocol, implausible eGFR values (<5 or >200 mL/min/1.73 m^2^), or those obtained following dialysis or kidney transplantation, and with serum creatinine concentrations <.2 or >15 mg/dL, were excluded from analyses. Progression of cardiomyopathy over time was assessed by left ventricular mass index (LVMI). LVMI was calculated using echocardiogram findings.^15^ Per protocol, implausible LVMI values (<10 or >150 g/m^2.7^) were excluded from analyses. For the progression of cardiomyopathy, patients were stratified by baseline left ventricular hypertrophy (LVH) defined as LVMI >48 g/m^2.7^ in female patients and >50 g/m^2.7^ in male patients.^16^, ^17^ In prespecified analyses, treated and untreated patients were stratified by classic or late‐onset (non‐classic) Fabry disease phenotype for all effectiveness outcomes.

Additional primary effectiveness endpoints were time to and age at a composite morbidity endpoint (comprising renal, cardiac or stroke events or death),^18^ and time to, and age at, death (survival analysis).^5^

Primary safety endpoints were the incidence of treatment‐emergent adverse events (TEAEs), serious adverse events (AEs) and infusion‐related reactions (IRRs). IRRs were defined as AEs that occurred during infusion or in the 24 h after infusion, and that were considered at least possibly related to agalsidase alfa treatment by the investigator. The severity and relatedness of AEs were also assessed. Data on the number of hospitalizations, reasons for hospitalizations, length of hospital stay and treatment received were collected as part of the healthcare utilization analysis. Investigation of drug‐specific antibodies was at the discretion of physicians, and the assessments were performed in a central laboratory.

### 2.3 Statistical analyses

There was no predetermined sample size for FOS, and all data collected from the time of consent to the time of withdrawal or database close were used. Baseline was defined as the closest value to (within 6 months before and 3 months after) the start of agalsidase alfa treatment or enrolment into the registry for untreated patients. Follow‐up was defined as the time from the first to the last recorded treatment or latest follow‐up in FOS. Descriptive statistics were reported for continuous and categorical variables; all confidence intervals were two‐sided, with the level of significance set at .05.

Changes in renal function and cardiomyopathy were presented as the mean annual rate of change in eGFR and LVMI, respectively, calculated using the interaction model procedure PROC MIXED in SAS/STAT^®^ version 9.4 (SAS Institute Inc., Cary, NC, USA). Mixed‐effects linear models were performed using an unstructured covariance structure and included the fixed effect of treatment status (treated or untreated), random effects of patients and time, and treatment‐by‐time interaction. Data were included for each patient if baseline and at least two post‐baseline measurements were available.

Kaplan–Meier time to morbidity analyses were conducted from treatment start to first composite event, with censoring at the patient's last visit. Kaplan–Meier survival analyses were conducted from birth to age at death. Rates were adjusted for the reducing number of patients over time, including drop‐outs and transfers.

## 3 RESULTS

### 3.1 Demographics and baseline clinical characteristics

In total, 4016 adult patients from 109 sites in 24 countries were enrolled in FOS (Table S1). Overall, 1864 adults (male, *n =* 957; female, *n =* 907) were treated with agalsidase alfa only, and 1613 adults (male, *n =* 378; female, *n =* 1235) were untreated (Table 1). Consistent with the approved indication, the median dose of agalsidase alfa for treated patients was .2 mg/kg, with nearly all patients having a treatment frequency of 11–18 days (Table S2). Treated patients had received agalsidase alfa for up to 21.6 years, with a median (minimum, maximum) time on treatment of 6.0 (0, 21.6) years. The treated population was markedly different to the untreated population at baseline (Table 1; Appendix S1); hence, formal comparisons between treated and untreated patients were not made. For example, the treated group included a greater proportion of patients with classic disease (61.4% vs. 29.6%) and more severe disease. The proportion of treated patients with the N215S genotype was greater among male than female patients (6.1% vs. 1.9%; Table 1). Reasons for discontinuation from FOS are presented in Table S3.

**TABLE 1:** Baseline demographics and clinical characteristics of patients treated with agalsidase alfa only and untreated patients enrolled in FOS.

Demographic/characteristic	Treated patients			Untreated patients		
	Male (*n* = 957)	Female (*n* = 907)	Total (*N* = 1864)	Male (*n* = 378)	Female (*n* = 1235)	Total (*N* = 1613)
Race, *n* (%)						
White	594 (63.0)	619 (69.9)	1213 (66.4)	256 (69.8)	803 (67.4)	1059 (67.9)
Asian	322 (34.1)	234 (26.4)	556 (30.4)	94 (25.6)	325 (27.3)	419 (26.9)
Black or African American	2 (.2)	1 (.1)	3 (.2)	1 (.3)	1 (.1)	2 (.1)
Other	25 (2.7)	31 (3.5)	56 (3.1)	16 (4.4)	63 (5.3)	79 (5.1)
*n* (*n* missing)	943 (14)	885 (22)	1828 (36)	367 (11)	1192 (43)	1559 (54)
Age at onset of symptoms, median (min, max) years	14.0 (.0, 72.0)	24.0 (.0, 71.0)	18.0 (.0, 72.0)	13.0 (.0, 76.0)	21.0 (.0, 75.0)	20.0 (.0, 76.0)
*n* (*n* missing)	657 (300)	518 (389)	1175 (689)	161 (217)	380 (855)	541 (1072)
Age at diagnosis, median (min, max), years	33.0 (.0, 78.0)	43.0 (.0, 80.0)	39.0 (.0, 80.0)	40.0 (2.0, 84.0)	35.0 (.0, 94.0)	36.0 (.0, 94.0)
*n* (*n* missing)	921 (36)	863 (44)	1784 (80)	353 (25)	1118 (117)	1471 (142)
Age at baseline, median (min, max), years	40.3 (18.0, 79.5)	49.5 (18.0, 81.5)	45.0 (18.0, 81.5)	45.0 (18.2, 85.1)	39.7 (18.0, 94.7)	40.6 (18.0, 94.7)
*n* (*n* missing)	957 (0)	907 (0)	1864 (0)	378 (0)	1235 (0)	1613 (0)
Age at last visit,^a^ median (min, max), years	53.6 (22.0, 92.2)	61.0 (22.3, 94.5)	57.1 (22.0, 94.5)	50.8 (20.0, 88.1)	46.2 (20.0, 97.7)	47.4 (20.0, 97.7)
*n* (*n* missing)	957 (0)	907 (0)	1864 (0)	378 (0)	1235 (0)	1613 (0)
Phenotype/genotype, *n* (%)						
Classic	231 (56.6)	278 (66.0)	509 (61.4)	67 (24.4)	282 (31.1)	349 (29.6)
Non‐classic	84 (20.6)	35 (8.3)	119 (14.4)	93 (33.8)	340 (37.5)	433 (36.7)
N215S (non‐classic)	25 (6.1)	8 (1.9)	33 (4.0)	31 (11.3)	79 (8.7)	110 (9.3)
D313Y	1 (.2)	7 (1.7)	8 (1.0)	22 (8.0)	36 (4.0)	58 (4.9)
Genetic variant unknown, benign or nonpathogenic	8 (2.0)	20 (4.8)	28 (3.4)	35 (12.7)	61 (6.7)	96 (8.1)
No definite *GLA* gene variant found	52 (12.7)	62 (14.7)	114 (13.8)	20 (7.3)	86 (9.5)	106 (9.0)
Unknown	7 (1.7)	11 (2.6)	18 (2.2)	7 (2.5)	22 (2.4)	29 (2.5)
*n* (*n* missing)	408 (549)	421 (486)	829 (1035)	275 (103)	906 (329)	1181 (432)
Total FOS‐MSSI score,^b^ mean (SD)	17.60 (10.69)	15.09 (9.95)	16.39 (10.41)	14.43 (11.99)	9.17 (8.91)	10.41 (9.98)
*n* (*n* missing)	926 (31)	865 (42)	1791 (73)	369 (9)	1188 (47)	1557 (56)
eGFR at baseline (patients aged ≥16 years), mean (SD), mL/min/1.73 m^2^	96.13 (31.28)	92.04 (23.37)	94.05 (27.61)	n/a	n/a	99.04 (21.15)
*n* (*n* missing)	557 (400)	576 (331)	1133 (731)	n/a	n/a	1164 (449)
eGFR at baseline (patients aged ≥18 years), mean (SD), mL/min/1.73 m^2^	96.06 (31.27)	92.04 (23.37)	94.01 (27.60)	91.68 (24.62)	100.99 (19.63)	99.01 (21.13)
*n* (*n* missing)	556 (401)	576 (331)	1132 (732)	248 (130)	915 (320)	1163 (450)
eGFR category at baseline, *n* (%)						
<60 mL/min/1.73 m^2^	78 (14.0)	61 (10.6)	139 (12.3)	28 (11.3)	27 (3.0)	55 (4.7)
≥60 mL/min/1.73 m^2^	478 (86.0)	515 (89.4)	993 (87.7)	220 (88.7)	888 (97.0)	1108 (95.3)
*n* (*n* missing)	556 (401)	576 (331)	1132 (732)	248 (130)	915 (320)	1163 (450)
LVMI at baseline, mean (SD), g/m^2.7^	61.07 (26.06)	55.29 (23.54)	58.25 (25.01)	50.85 (23.90)	36.29 (15.30)	39.40 (18.47)
*n* (*n* missing)	328 (629)	313 (594)	641 (1223)	160 (218)	589 (646)	749 (864)
LVH status at baseline, *n* (%)						
LVH	195 (59.5)	169 (54.0)	364 (56.8)	65 (40.6)	93 (15.8)	158 (21.1)
No LVH	133 (40.5)	144 (46.0)	277 (43.2)	95 (59.4)	496 (84.2)	591 (78.9)
*n* (*n* missing)	328 (629)	313 (594)	641 (1223)	160 (218)	589 (646)	749 (864)

*Note*: Unless specified otherwise, adults were defined as patients aged ≥18 years at baseline. Baseline is defined as the value closest (within 66 months before to 33 months after) to the earliest start of treatment or enrolment into FOS in untreated patients.

Abbreviations: eGFR, estimated glomerular filtration rate; FOS, Fabry Outcome Survey; FOS‐MSSI, FOS adjusted Mainz Severity Index Score; *GLA*, alpha galactosidase A gene; LVH, left ventricular hypertrophy; LVMI, left ventricular mass index; min, minimum; max, maximum; SD, standard deviation.

^a^ Before database lock.

^b^ Disease severity is assessed by the total FOS‐MSSI score obtained: mild (0 to 18), moderate (19 to 38) or severe (39 to 65).

### 3.2 Effectiveness outcomes

#### 3.2.1 Renal function

**FIGURE 1:**
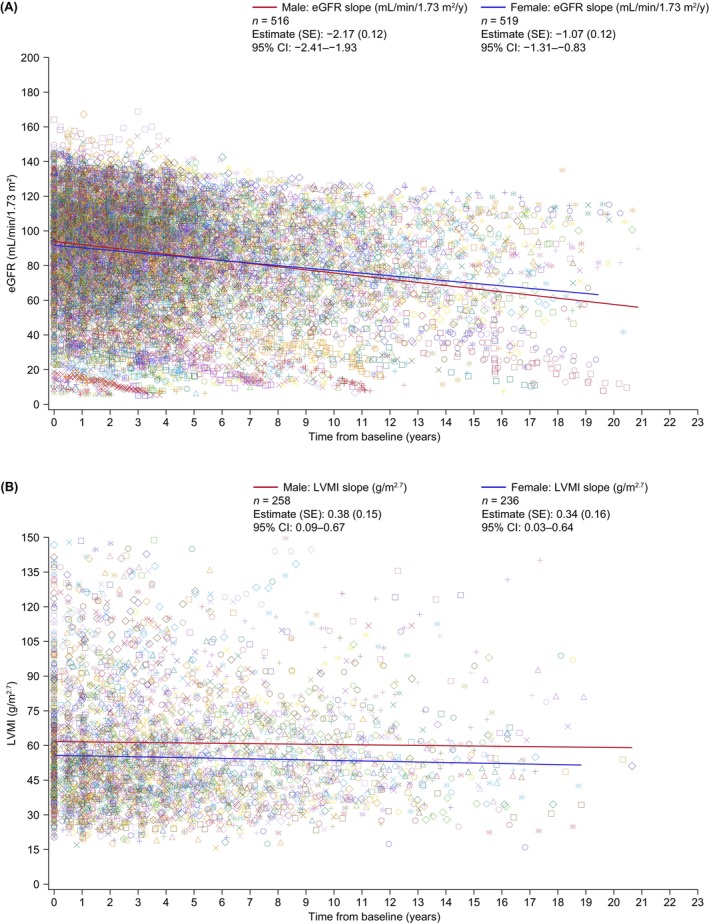
Clinical outcomes in adults with Fabry disease in FOS treated with agalsidase alfa only stratified by sex: Mean annualized change in eGFR^a^ (A) and in LVMI (B). The model includes sex as a fixed effect and random intercept, and time effects, adjusting for covariates of age and baseline eGFR or LVMI. A sex‐by‐time interaction term was also included. Random effects were assumed to have an unstructured covariance structure within each patient covariance structure. eGFR values outside the range 5–200 mL/min/1.73 m^2^ and LVMI values outside the range 10–150 g/m^2.7^ were excluded. Baseline is defined as the value closest (within 6 months before to 3 months after) to the start of treatment. CI, confidence interval; eGFR, estimated glomerular filtration rate; FOS, Fabry Outcome Survey; LVMI, left ventricular mass index; SE, standard error. ^a^Data are presented for patients aged ≥18 years (post hoc analysis).

The annualized changes in eGFR slope (standard error; SE) in males and females aged ≥18 years treated with agalsidase alfa only were −2.17 (.12) and −1.07 (.12) mL/min/1.73 m^2^, respectively (Table 2; Figure 1A). In treated males and females with normal or stage 2 renal function at baseline (≥60 mL/min/1.73 m^2^), the annualized decline was −1.97 (.16) and −.99 (.10) mL/min/1.73 m^2^, respectively. Declines in eGFR were more pronounced in those with poor renal function at baseline (<60 mL/min/1.73^2^: males, −3.60 [.43]; females, −1.17 [.28]) (Table 2). Similar findings were observed for patients with proteinuria at baseline (Table 2) and for treated patients aged ≥16 years, which was the primary population for this endpoint (males, −2.20 [.12] mL/min/1.73 m^2^, *n =* 542; females, −1.04 [.12] mL/min/1.73 m^2^, *n =* 531). In treated males aged ≥18 years with classic or non‐classic Fabry disease, the mean eGFR slope (SE) was −2.31 (.27) and −1.28 (.36) mL/min/1.73 m^2^/year, respectively. The corresponding values for treated females were −1.06 (.18) and −.55 (.42) mL/min/1.73 m^2^/year, respectively (Table 2).

**TABLE 2:** Progression of renal disease in adults with Fabry disease treated with agalsidase alfa only in FOS (post hoc analysis).

Annual rate of change in eGFR, slope (SE), mL/min/1.73 m^2^	Male	Female
Treated patients	*n* = 516 −2.17 (.12)	*n* = 519 −1.07 (.12)
Baseline eGFR, mL/min/1.73 m^2^		
<60	*n* = 70 −3.60 (.43)	*n* = 56 −1.17 (.28)
≥60	*n* = 446 −1.97 (.16)	*n* = 463 −.99 (.10)
Baseline urinary protein level, g/24 h^a^		
≥1.0	*n* = 57 −3.28 (.41)	*n* = 33 −1.39 (.36)
≥.3 to <1.0	*n* = 78 −1.71 (.32)	*n* = 55 −.53 (.28)^b^
<.3	*n* = 159 −.22 (.25)^c^	*n* = 190 −.03 (.16)^d^
*n* missing	222	241
Phenotype		
Classic	*n* = 114 −2.31 (.27)	*n* = 147 −1.06 (.18)
Non‐classic (includes N215S)	*n* = 72 −1.28 (.36)	*n* = 24 −.55 (.42)
*n* missing	330	348

*Note*: Adults were defined as patients aged ≥18 years at baseline. The annual rate of change within each sex was calculated using the Wald test. The annual rate of change for each subgroup within each sex was statistically significant (*p* < .0001), except for those subgroups indicated separately.

Abbreviations: eGFR, estimated glomerular filtration rate; FOS, Fabry Outcomes Survey; SE, standard error.

^a^ Proteinuria at baseline is defined as ≥1.0 g/24 h.

^b^ Slope of annual rate of change was not significant (*p* = .0592).

^c^ Slope of annual rate of change was not significant (*p* = .3907).

^d^ Slope of annual rate of change was not significant (*p =* .8535).

Annualized changes in eGFR slope were similar for untreated males and untreated females, but lower than those reported in treated patients (Appendix S1).

Fabry disease gene variant classification and use of angiotensin‐converting enzyme inhibitors or angiotensin receptor blockers are presented by baseline urinary protein level subgroups for each sex in Table S4.

#### 3.2.2 Cardiomyopathy

The estimated annualized change in LVMI (slope [SE]) in adults treated with agalsidase alfa was .38 (.15) for males and .34 (.16) g/m^2.7^ for females (Table 3; Figure 1B). In treated males and females with LVH at baseline, annualized changes were .40 (.19) and .30 (.22) g/m^2.7^, respectively; for those without LVH at baseline, corresponding values were .26 (.23) for males and .51 (.24) g/m^2.7^ for females (Table 3). Changes in LVMI for classic and non‐classic disease are presented in Table 3. Estimated annualized changes from baseline in LVMI (SE) for untreated males (*n =* 40) and females (*n =* 178) were not significant −.45 (.33) g/m^2.7^ and −.03 (.14) g/m^2.7^, respectively.

**TABLE 3:** Progression of cardiac disease in adults with Fabry disease treated with agalsidase alfa only in FOS.

Annual rate of change in LVMI, slope (SE), g/m^2.7^	Men	Women
Treated patients	*n* = 258 .38 (.15)	*n =* 236 .34 (.16)
LVH at baseline		
Yes	*n* = 157 .40 (.19)	*n =* 136 .30 (.22)^a^
No	*n* = 101 .26 (.23)^b^	*n =* 100 .51 (.24)
Phenotype		
Classic	*n* = 49 .58 (.27)	*n =* 66 .35 (.23)^c^
Non‐classic (includes N215S)	*n* = 46 .76 (.34)	*n* = 17 .70 (.45)^d^

*Note*: The annual rate of change within each sex was calculated using the Wald test. The annual rate of change for each subgroup within each sex was statistically significant (*p* < .05), except for those subgroups indicated separately.

Abbreviations: FOS, Fabry Outcomes Survey; LVH, left ventricular hypertrophy; LVMI, left ventricular mass index; SE, standard error.

^a^ Slope of annual rate of change was not significant (*p =* .1741).

^b^ Slope of annual rate of change was not significant (*p =* .2610).

^c^ Slope of annual rate of change was not significant (*p =* .1247).

^d^ Slope of annual rate of change was not significant (*p =* .1196).

#### 3.2.3 Morbidity

**FIGURE 2:**
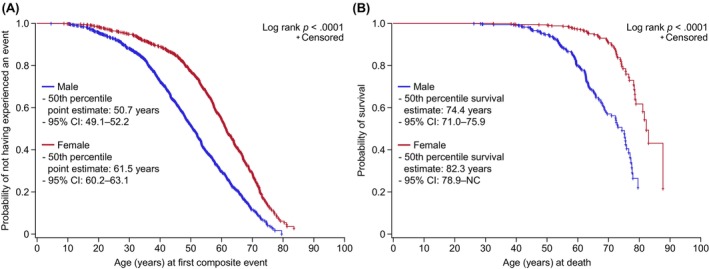
Clinical outcomes in adults with Fabry disease in FOS treated with agalsidase alfa only stratified by sex: Age at which 50% of treated men and women had experienced their first composite morbidity event (renal, cardiac or stroke event, or death) (A) and probability of survival with age (B). Baseline is defined as the value closest (within 6 months before to 3 months after) to the earliest start of treatment with agalsidase alfa. Kaplan–Meier analyses were conducted with censoring at patient's latest visit in FOS. CI, confidence interval; FOS, Fabry Outcome Survey; NC, not calculable.

A total of 57.5% (636/1106) and 49.5% (484/977) of treated males and females, respectively, had at least one composite event; the median (minimum, maximum) age at first composite event was 41.2 (4.5, 79.5) and 52.2 (7.8, 85.1) years, respectively. The median (95% confidence interval [CI]) time from treatment initiation to first composite event was 56.3 (45.6–66.7) and 83.4 (65.7–98.0) months in males and females, respectively. The estimated (95% CI) age at which 50% of the treated group had experienced a first composite event was 50.7 (49.1–52.2) years in males and 61.5 (60.2–63.1) years in females (Figure 2A). Morbidity data by disease phenotype for treated patients are presented in Table S5.

Overall, 33.0% (148/448) and 19.5% (272/1392) of untreated males and females, respectively, had at least one composite event. The median age at first composite event was similar for untreated and treated males, but approximately 10 years lower for untreated than treated females (Appendix S1).

#### 3.2.4 Mortality

Among treated patients, 151 (13.7%) males and 60 (6.1%) females died during the follow‐up period; median (minimum, maximum) ages at death were 59.1 (26.2, 79.6) years and 69.9 (32.5, 87.7) years, respectively. The estimated (95% CI) age at which 50% of treated males and females died was 74.4 (71.0–75.9) years and 82.3 (78.9, not calculable) years, respectively (Figure 2B). The estimated (95% CI) probability of surviving for at least 10 years, 15 years and 19 years from treatment initiation was .863 (.834–.887), .760 (.714–.799) and .686 (.626–.738), respectively, for treated males, and was .931 (.906–.950), .883 (.843–.914) and .773 (.647–.859), respectively, for treated females. Mortality data for the treated group stratified by classic and non‐classic disease are shown in Table S6.

In the untreated group, 20 (4.5%) males and 13 (.9%) females died during the follow‐up period. Median ages at death were more than 6 years higher for the untreated than for the treated patients (Appendix S1).

### 3.3 Safety outcomes

Overall, 7378 TEAEs were reported for 1083 patients (male, 603; female, 480) treated with agalsidase alfa only, representing 58.1% of the total treated population (male, 63.0%; female, 52.9%; Table 4). In total, 18.7% of TEAEs were considered related to agalsidase alfa treatment by investigators. TEAEs were mild (27.4% of events reported in 8.1% of patients) or moderate (21.5% of events reported in 12.7% of patients) in severity. The most common TEAEs were headache and diarrhoea (4.5% of patients each; Table S7). Drug‐specific antibodies were associated with 22 events in 21 patients (1.1%) 17 events in 16 (1.7%) males and two events in two (.2%) females were considered IRRs. Event severity was reported for one patient (.1%; recorded as mild [<.1%]). Three male patients (.2%) developed neutralizing antibodies considered possibly (*n* = 2) or probably (*n* = *1*) related to treatment; all three were considered moderate in severity.

**TABLE 4:** Summary of TEAEs in adults with Fabry disease treated with agalsidase alfa in FOS.

TEAE category, *n* (%) [m] [%]	Male (*n =* 957)	Female (*n =* 907)	Total (*N =* 1864)
TEAEs	603 (63.0) [4742] [100]	480 (52.9) [2636] [100]	1083 (58.1) [7378] [100]
TEAEs by severity			
Not recorded	152 (15.9) [1857] [39.2]	113 (12.5) [883] [33.5]	265 (14.2) [2740] [37.1]
Mild	76 (7.9) [1209] [25.5]	75 (8.3) [814] [30.9]	151 (8.1) [2023] [27.4]
Moderate	103 (10.8) [990] [20.9]	133 (14.7) [599] [22.7]	236 (12.7) [1589] [21.5]
Severe	132 (13.8) [182] [10.6]	101 (11.1) [267] [10.1]	233 (12.5) [771] [10.4]
Fatal^a^	140 (14.6) [182] [3.8]	58 (64.) [73] [2.8]	198 (10.6) [255] [3.5]
TEAEs by relationship to treatment			
Not recorded	3 (.3) [57] [1.2]	5 (.6) [51] [1.9]	8 (.4) [108] [1.5]
Not related	422 (44.1) [3759] [79.3]	370 (40.8) [2240] [85.0]	792 (42.5) [5999] [81.3]
Possibly related	93 (9.7) [395] [8.3]	63 (6.9) [236] [9.0]	156 (8.4) [631] [8.6]
Probably related	85 (8.9) [531] [11.2]	42 (4.6) [109] [4.1]	127 (6.8) [640] [8.7]
TEAEs by seriousness			
Not recorded	1 (.1) [18] [.4]	1 (.1) [27] [1.0]	2 (.1) [45] [.6]
Serious	421 (44.0) [1543] [32.5]	298 (32.9) [868] [32.9]	719 (38.6) [4922] [32.7]
Non‐serious	181 (18.9) [3181] [67.1]	181 (20.0) [1741] [66.0]	362 (19.4) [2411] [66.7]
Treatment‐emergent IRR	153 (16.0) [778] [16.4]	88 (9.7) [229] [8.7]	241 (12.9) [1007] [13.6]

*Note*: Adults were defined as patients aged ≥18 years at the time of the cut‐off date. Percentage of patients is based on the total number of adult treated patients. Percentage of adverse events is based on the total number of events experienced. Events that could not be classified as an IRR (‘Yes’ or ‘No’) owing to lack of information were imputed as ‘No’.

Abbreviations: (%), percentage of patients; [%], percentage of adverse events; FOS, Fabry Outcome Survey; IRR, infusion‐related reaction; n, number of patients; [m], number of adverse events; TEAE, treatment‐emergent adverse event.

^a^ Values for the number of fatal TEAEs differ from those reported in the Mortality section because not all deaths were reported as TEAEs in the database.

There were 2411 treatment‐emergent serious AEs (SAEs) (including fatal events) in 719 patients treated with agalsidase alfa only (male, 421; female, 298), representing 38.6% of the total treated population (male, 44.0%; female, 32.9%; Table 5). The most common treatment‐emergent SAEs (excluding fatal SAEs) per System Organ Class were cardiac disorders (male, 15.4%; female, 11.0% reported at least one event) and nervous system disorders (male, 10.6%; female, 9.8% reported at least one event; Table S8). The majority of treatment‐emergent SAEs (95.3%) were classified as not related to treatment by investigators. SAEs severity was reported as ‘moderate’ for 36.0% of events (867/2411, reported in 8.6% of patients) and ‘severe’ for 28.8% of events (695/2411, reported in 11.5% of patients). A total of 201 (10.8%) treated patients died (male, 143; female, 58; including three deaths not reported as TEAEs). Treatment‐emergent SAEs are reported in Table 5. The most common causes of death (known for 130/201) in the overall treated population were cardiovascular disease, stroke and myocardial infarction. There were 33 deaths among untreated patients during the follow‐up period (19 of which were reported as SAEs). The most common causes of death (known for 16/33) in untreated patients were cardiovascular disease, respiratory disease and stroke.

**TABLE 5:** Summary of treatment‐emergent SAEs in adults with Fabry disease treated with agalsidase alfa in FOS.

TEAE category, *n* (%) [m] [%]	Male (*n =* 957)	Female (*n =* 907)	Total (*N =* 1864)
Treatment‐emergent SAEs	421 (44.0) [1543] [100]	298 (32.9) [868] [100]	719 (38.6) [2411] [100]
Treatment‐emergent SAEs by severity			
Not recorded	65 (6.8) [250] [16.2]	38 (4.2) [100] [11.5]	103 (5.5) [350] [14.5]
Mild	20 (2.1) [138] [8.9]	24 (2.6) [106] [12.2]	44 (2.4) [244] [10.1]
Moderate	77 (8.0) [527] [34.2]	83 (9.2) [340] [39.2]	160 (8.6) [867] [36.0]
Severe	119 (12.4) [446] [28.9]	95 (10.5) [249] [28.7]	214 (11.5) [695] [28.8]
Fatal^a^	140 (14.6) [182] [11.8]	58 (6.4) [73] [8.4]	198 (10.6) [255] [10.6]
Treatment‐emergent SAEs by relationship to treatment			
Not recorded	1 (.1) [18] [1.2]	3 (.3) [9] [1.0]	4 (.2) [27] [1.1]
Not related	385 (40.2) [1469] [95.2]	274 (30.2) [828] [95.4]	659 (35.4) [2297] [95.3]
Possibly related	25 (2.6) [40] [2.6]	19 (2.1) [29] [3.3]	44 (2.4) [69] [2.9]
Probably related	10 (1.0) [16] [1.0]	2 (.2) [2] [.2]	12 (.6) [18] [.7]
Treatment‐emergent serious IRRs	18 (1.9) [28] [1.8]	16 (1.8) [25] [2.9]	34 (1.8) [53] [2.2]
Treatment‐emergent SAEs leading to death	140 (14.6)	58 (6.4)	198 (10.6)
Treatment‐emergent SAEs leading to death by relationship to treatment			
Not recorded	3 (.3)	2 (.2)	5 (.3)
Not related	132 (13.8)	56 (6.2)	188 (10.1)
Possibly related^b^	5 (.5)	0 (.0)	5 (.3)

*Note*: Adults were defined as patients aged ≥18 years at the time of the TEAE. Percentage of patients is based on the total number of adult treated patients. Percentage of adverse events is based on the total number of events experienced. Events that could not be classified as an IRR (‘Yes’ or ‘No’) owing to lack of information were imputed as ‘No’.

Abbreviations: (%), percentage of patients; [%], percentage of adverse events; FOS, Fabry Outcome Survey; IRR, infusion‐related reaction; n, number of patients; [m], number of adverse events; SAE, serious adverse event; TEAE, treatment‐emergent adverse event.

^a^ Data for the number of fatal TEAEs differs from those reported in the Mortality section because three deaths were not recorded as TEAEs in the database.

^b^ Treatment‐emergent SAEs resulting in death in five patients (.3%; all male) were considered by investigators as possibly related to treatment: a 59‐year‐old with septic shock, abnormal hepatic function, decreased platelet count and cerebral infarction; a 59‐year‐old with pulmonary oedema; a 61‐year‐old with acute cardiac failure and pneumonia; a 32‐year‐old who had sudden death (unknown cause); and a 74‐year‐old with arrhythmia. An association of the deaths to treatment could not be established.

Event rates of dialysis and kidney transplantation related to Fabry disease are presented in Table S9. A summary of the TEAEs occurring during or after agalsidase alfa treatment at home is reported in Appendix S1 and Table S10; the clinical characteristics of patients who received therapy at home at least once are presented in Table S11.

Overall, 1007 treatment‐emergent IRRs were reported by 241 patients (male, 153; female, 88), representing 12.9% of the total treated population (male, 16.0%; female, 9.7%; Table S12). The most commonly recorded severity category of IRRs was ‘mild’, with 24.7% of events reported in 4.1% of patients, followed by ‘moderate’, with 12.4% of events reported in 2.8% of patients.

### 3.4 Healthcare resource utilization

Overall, 595 treated patients were admitted to the hospital during the follow‐up period. The most common reasons for hospitalization (where known) were surgery, cardiovascular disease and infections (Table S13).

## 4 DISCUSSION

Here we provide real‐world data on adults from FOS on the natural history of Fabry disease and the long‐term effectiveness and safety of enzyme replacement therapy with agalsidase alfa from over two decades (2001–2022). In all, 1864 adults (aged ≥18 years) were treated with agalsidase alfa only, and 1613 adults did not receive Fabry disease‐specific treatment.

Among patients who received agalsidase alfa only, annualized reductions from baseline in renal function (as measured by eGFR) were relatively stable in females and declined slightly in males; the declines were more pronounced for males with poor renal function and proteinuria at baseline. Annualized changes in LVMI remained stable and were similar for treated males and females. In patients with LVH at baseline, annualized increases in LVMI were slightly higher for males with LVH than for those without LVH, whereas increases among females with LVH were lower than for those without LVH. In the treated group, females reached the first composite morbidity event (renal, cardiac or stroke event, or death) at an older age than males, and mean age at death was substantially lower for male patients. Agalsidase alfa treatment was generally well tolerated, with a safety profile consistent with previous reports.^17^, ^18^, ^19^, ^20^

Long‐term data on Fabry disease‐related outcomes in untreated populations have been described previously.^17^, ^18^, ^19^, ^20^ Untreated patients in FOS showed slow disease progression over time, with worse outcomes for males than females, as seen for treated patients. The untreated group generally had more favourable outcomes, which likely reflects the milder disease severity of this population at FOS enrolment compared with treated patients. As previously discussed, this difference, as well as those in follow‐up between treated and untreated patients, precludes formal comparisons between these populations.^5^, ^21^

In patients treated with agalsidase alfa in FOS, renal and cardiac disease progression (assessed as rate of change of eGFR and LVMI, respectively), was slower than that reported previously for the natural history of the disease.^17^, ^19^ As previously reported,^7^, ^22^, ^23^, ^24^ renal decline was more pronounced in those with poorer renal function at baseline and in males with classic Fabry disease. The annualized change in eGFR in treated male and female adults (aged ≥18 years) with eGFR ≥60 mL/min/1.73 m^2^ was −1.97 and −.99 mL/min/1.73 m^2^, respectively. Reductions in eGFR in females were broadly consistent with those previously reported for the general adult population without Fabry disease aged >40 years (−1.0 mL/min/1.73 m^2^).^25^ For males and females with eGFR <60 mL/min/1.73 m^2^, the eGFR slope was −3.60 and −1.17 mL/min/1.73 m^2^, respectively. A retrospective chart review (between 1944 and 2002) of 447 patients with Fabry disease,^19^ assessed the mean rate of eGFR decline in untreated patients. For males and females with eGFR ≥60 mL/min/1.73 m^2^, it was −3.0 and −.9 mL/min/1.73 m^2^/year, respectively, and for those with eGFR <60 mL/min/1.73 m^2^ it was −6.8 and −2.1 mL/min/1.73 m^2^/year, respectively. Advances in healthcare since that study^19^ may explain some of these differences. An analysis of data for untreated patients from the natural history cohort^19^ compared with agalsidase beta‐treated patients from the Fabry Registry (matched 1:1 based on sex, phenotype, age and baseline eGFR) reported similar declines in overall eGFR slopes (−3.2 and −1.5 mL/min/1.73 m^2^/year, respectively).^26^

In the FOS analysis of progression of cardiomyopathy, the annualized change in LVMI in males and females receiving agalsidase alfa was .38 and .34 g/m^2.7^, respectively. Kampmann et al.^17^ assessed changes in LVMI index in 78 untreated patients with Fabry disease and reported an average rate of increase of 4.07 and 2.31 g/m^2.7^ per year in males and females, respectively. Overall, these findings are consistent with the previously reported beneficial effects of agalsidase alfa on renal function and cardiomyopathy in Fabry disease.^4^, ^27^ A non‐significant reduction from baseline in LVMI was observed among untreated males, which is unlikely to be attributable to changes associated with age^28^ or Fabry disease progression. However, the standards of care for Fabry disease have improved, with patients being diagnosed sooner and treatment started earlier than before, which means that LVMI may be lower on average at enrolment to FOS.

Treatment with agalsidase alfa in FOS also appeared to have a beneficial effect on morbidity and mortality. Approximately half of treated males and females had at least one composite morbidity event over a median of 6 years of treatment. Previous studies of Fabry disease treatments (agalsidase alfa, agalsidase beta and/or migalastat) have reported incidences of composite morbidity outcomes of 17%–37% of patients over 1.5–10 years.^18^, ^29^, ^30^, ^31^, ^32^, ^33^, ^34^, ^35^, ^36^, ^37^ However, these studies used different definitions of composite clinical outcomes, methods of analyses and durations of treatment, so they cannot be directly compared.^33^ In the present analyses, the proportion of untreated female patients was markedly larger than that of male patients, as would be expected given that Fabry disease typically presents as more severe and progressive forms in male patients.^38^ Indeed, some guidelines recommend that, although disease‐specific treatment should be initiated as early as possible in male patients with classic disease, initiation in female patients should be guided by indicators of organ damage.^39^ It was striking, therefore, that the estimated age at first composite event was younger for untreated than for treated female patients in our dataset. This may highlight the importance of timely treatment initiation in female patients, and underscore the need for improved disease diagnosis, monitoring and treatment. Time to first composite morbidity event was longer for female than male patients. This may reflect the fact that male patients typically have more severe and progressive forms of the disease.^23^, ^40^ Indeed, in FOS, the proportion of patients with the N215S genotype, which is associated with severe cardiac manifestations,^41^ was greater for male than female patients (6.1% vs. 1.9%). In the untreated patients characterized by Schiffmann et al.,^19^ half of the male and female patients had a composite morbidity event (renal, cardiac or stroke event, or death) by 41 years and 53 years, respectively. In FOS, the estimated age at which 50% of treated patients had experienced a composite morbidity event was higher in female patients (61.5 years) than in male patients (50.7 years). Furthermore, male patients treated with agalsidase alfa in FOS had a longer survival time (50% survival at 74.4 years of age) than the untreated male population^19^ (50% survival at 60 years of age).

TEAEs were reported in 58.1% of treated patients in FOS and were consistent with the known safety profile of agalsidase alfa and clinical manifestations of Fabry disease. Most TEAEs were mild or moderate in severity and were not considered related to agalsidase alfa. Overall, 201 (10.8%) adults receiving agalsidase alfa died during follow‐up. The aetiology of deaths and other SAEs was consistent with the cardiac, renal and cerebrovascular organ involvement typically associated with Fabry disease.^3^, ^19^ Approximately half of treated patients received agalsidase alfa at home; in these patients, the incidence of TEAEs (53.6%) was similar to that for the overall treated population, suggesting that home therapy is not associated with additional safety risks.

Analyses of FOS data have vastly contributed to a better understanding of Fabry disease and have informed the management of patients with this rare disease.^4^ Owing to the retrospective nature of the FOS registry, assessments were completed according to the routine care provided to patients, and in some cases, data were missing, incomplete or collected inconsistently. Therefore, an obvious key limitation of these analyses is that they could only be performed on a subset of patients for whom relevant data were available. Similarly, these analyses focus on patients who were treated with agalsidase alfa only (treated patients) and those who never received disease‐specific treatment (untreated patients). Hence, the data do not fully reflect the general population in clinical practice, where patients may switch from one treatment to another. It may also have been of interest to assess data on the levels of globotriaosylsphingosine (lyso‐Gb3) in terms of its relationship to disease burden, clinical course and therapeutic response.^42^ However, data on lyso‐Gb3 were only recorded in FOS from 2016, and, hence, insufficient data were available to support informative analyses. Similarly, further assessment of concomitant medications taken by patients (such as anti‐thrombotic therapies, given that cerebrovascular events are a major cause of morbidity and mortality in Fabry disease^4^) and of broader cardiac outcomes (including cardiac fibrosis and ECG recordings) would have been informative, but were not possible owing to a paucity of robust data. In addition, evolution in the standards of Fabry disease diagnosis and care over time may have affected the results. There is also a possibility of patient enrolment bias, and the registry data may, therefore, not be generalizable to all patients with Fabry disease. Similarly, these analyses did not follow‐up patients who discontinued treatment; hence, there is potential for bias in terms of understanding the effects of agalsidase alfa after treatment discontinuation.

In conclusion, data collected over the 20 years of the FOS registry demonstrate the effectiveness and safety of agalsidase alfa in adults with Fabry disease. Treatment with agalsidase alfa appeared to slow renal deterioration and progression of cardiomyopathy compared with the published natural history of the disease, with no new safety signals identified.

## AUTHOR CONTRIBUTIONS

DAH, GP‐M, CK, CA, JB, SJ, KN, DM‐N, RR, MLW, JS, UR and RG each provided substantial contributions to the design of this study and the interpretation of literature for the work; were involved in drafting the work or revising it critically for important intellectual content; approved the final version to be published; and have agreed to be accountable for all aspects of the work in ensuring that questions related to the accuracy or integrity of any part of the work are appropriately investigated and resolved. All authors read and approved the final manuscript.

## FUNDING INFORMATION

FOS is funded by Takeda Pharmaceuticals International AG. Collection and analysis of data in FOS are supported by Takeda. Medical writing and editorial support were provided by Tove Anderson, PhD, of PharmaGenesis London, London, UK, and funded by Takeda Development Center Americas, Inc. The final decision to submit the manuscript for publication was made by the authors.

## CONFLICT OF INTEREST STATEMENT

DAH reports honoraria from AceLink Therapeutics, Amicus Therapeutics, Chiesi, Freeline Therapeutics, Idorsia, Protalix, Sanofi Genzyme and Takeda. She is a member of the FOS Steering Committee. GP‐M reports honoraria from Alexion, Amicus Therapeutics, BioMarin Pharmaceutical, Chiesi, Kyowa Kirin, Lucane Pharma, Sanofi and Takeda, and reports unrestricted grants from Sanofi and Takeda to the Vall d'Hebron Institute of Research Foundation for funding research on rare diseases. He is a member of the FOS Steering Committee. CK reports honoraria for speaking and/or advisory boards from Amicus Therapeutics, BioMarin Pharmaceutical, Gore and Takeda. He is a member of the FOS Steering Committee. CA is a former employee of Takeda Pharmaceuticals International AG and a current employee of Medison Pharma, Switzerland. JB is an employee of Takeda Pharmaceuticals International AG and a stockholder of Takeda Pharmaceuticals Company Limited. SJ is an employee of Takeda Development Center Americas, Inc. and a stockholder of Takeda Pharmaceuticals Company Limited. KN reports honoraria from Amicus Therapeutics, Sanofi Genzyme and Takeda. She is a member of the FOS Steering Committee. D‐MN reports honoraria and speaker fees from Sanofi Genzyme and Takeda, and reports research grants from BioMarin Pharmaceutical, Sanofi Genzyme and Takeda. He is a member of the FOS Steering Committee. RR reports honoraria, speaker fees and consulting fees from Amicus Therapeutics, CSL Behring, Gador, Novartis, Sanofi Genzyme and Takeda. He is a member of the FOS Steering Committee. MLW reports grants, personal fees and travel support from Alexion, Amicus Therapeutics, Chiesi, Idorsia, Protalix, Sanofi and Takeda. He is a member of the FOS Steering Committee. JS is a former employee of Takeda Pharmaceuticals International AG and a current employee of KalVista Pharmaceuticals, Switzerland. UR reports honoraria for speaking and/or advisory boards from Amicus Therapeutics, Sanofi Genzyme and Takeda, and reports research grants from Amicus Therapeutics, IntraBio and Takeda. She is a member of the FOS Steering Committee. RG reports honoraria, consulting fees, speaker fees, research funding and/or travel reimbursement from Alexion, Alnylam Pharmaceuticals, Amicus Therapeutics, Astellas Pharma, Azafaros, BioMarin Pharmaceutical, Chiesi, JCR Pharmaceuticals, Novartis, Paradigm BioPharmaceuticals, Passage Bio, Praxis Precision Medicines, PTC Therapeutics, Regenxbio, Sanofi, Takeda and Ultragenyx. He is a member and the current chair of the FOS Steering Committee.

## Supporting information


Appendix S1.

## Data Availability

The data sets, including the redacted study protocol, redacted statistical analysis plan, and individual participants' data supporting the results reported in this article, will be made available within 3 months from initial request to researchers who provide a methodologically sound proposal. The data will be provided after its de‐identification, in compliance with applicable privacy laws, data protection and requirements for consent and anonymization.
