# Supplementary material for: Long‐term effectiveness and safety outcomes in adults with Fabry disease treated with agalsidase alfa: 20 years of data from the Fabry Outcome Survey
Source: Eur J Clin Invest. 2025 Nov 6;56(1):e70142. doi: 10.1111/eci.70142 (PMC12820918; doi:10.1111/eci.70142)
Supplement: Supplementary file 1 — Appendix S1. [file ECI-56-e70142-s001.docx]

**Supplementary Results**

**Long-term effectiveness and safety outcomes in adults with Fabry disease treated with agalsidase alfa: 20 years of data from the Fabry Outcome Survey**

**Running title: Adult data from FOS: two decades of experience**

Derralynn A. Hughes, FRCPath,^1^ Guillem Pintos-Morell, MD, PhD,^2,3^ Christoph Kampmann, MD,^4^ Christina Anagnostopoulou, MSc,^5a^ Jaco Botha, MSc,^5^ Siddharth Jain, MD,^6^ Kathleen Nicholls, MD,^7,8^ Dau-Ming Niu, MD,^9,10^ Ricardo Reisin, MD,^11^ Michael L. West, MD,^12^ Jörn Schenk, MD,^5b^ Uma Ramaswami, MD,^1^ Roberto Giugliani, MD^13^

^1^Royal Free London NHS Foundation Trust, University College London, London, UK

^2^Vall d’Hebron Research Institute, Barcelona, Spain

^3^Medical Consulting Committee, MPS-Lisosomales Association, Spain

^4^Johannes Gutenberg School of Medicine, University of Mainz, Mainz, Germany

^5^Takeda Pharmaceuticals International AG, Zurich, Switzerland

^6^Takeda Development Center Americas, Inc., Cambridge, Massachusetts, USA

^7^The Royal Melbourne Hospital, Parkville, Victoria, Australia

^8^University of Melbourne, Parkville, Victoria, Australia

^9^Institute of Clinical Medicine, National Yang-Ming Chiao Tung University, Taipei, Taiwan

^10^Taipei Veterans General Hospital, Taipei, Taiwan

^11^Hospital Británico de Buenos Aires, Buenos Aires, Argentina

^12^Department of Medicine, Dalhousie University, Halifax, Nova Scotia, Canada

^13^Department of Genetics, UFRGS, Medical Genetics Service, HCPA, IMASP, Dasa Genomics and Casa dos Raros, Porto Alegre, Brazil

^a^Affiliation at the time of the analysis was Takeda Pharmaceuticals International AG, Zurich, Switzerland; current affiliation is Medison Pharma, Zug, Switzerland.

^b^Affiliation at the time of the analysis was Takeda Pharmaceuticals International AG, Zurich, Switzerland; current affiliation is KalVista Pharmaceuticals, Zug, Switzerland.

**Assessments**

Fabry disease variants were assigned a phenotype based on published literature:^1-16^ the [fabry-database.org](https://url.uk.m.mimecastprotect.com/s/yt7mClRvPS2QpXDtGfNHzv7fR?domain=fabry-database.org) *GLA* variant database^17^ and the International Fabry Disease Genotype–Phenotype Database.^18^ Non-classic Fabry disease variants included N215S and IVS4+919G>A,^19^ but excluded D313Y,^20^ variants of unknown significance, those thought to be benign or non-pathogenic, and those for which no definite phenotype could be assigned.

**Demographics and baseline clinical characteristics**

*Baseline disease severity in treated patients*

Renal function at baseline was normal or in the early stages of decline (stage 2) for the majority of patients (87.7% [993/1132] of those with estimated glomerular filtration rate [eGFR] data available) who went on to receive agalsidase alfa. Cardiac involvement in the form of left ventricular hypertrophy (LVH) was already established at baseline in 56.8% (364/641) of patients with available data who received treatment. Disease severity at baseline was broadly similar in male and female patients who received treatment; however, the overall disease burden as measured by the Fabry Outcomes Survey adjusted Mainz Severity Score Index (FOS-MSSI) was slightly greater in male than female patients (17.60 vs. 15.09). Male patients in the treated group were younger than female patients at symptom onset, diagnosis and treatment start (by about 10 years; **Table 1**).

*Baseline disease severity in untreated patients*

Renal function at baseline was also normal or in the early stages of decline (stage 2) in most untreated patients (95.3% [1108/1163] of those with eGFR data available). Cardiac involvement in the form of LVH was already established in 21.1% (158/749) of untreated patients with available data. Disease severity at baseline was more pronounced in untreated male than female patients, including when measured by the FOS-MSSI (14.43 vs. 9.17, respectively). Male patients in the untreated group were younger than female patients at symptom onset (by approximately 2 years; **Table 1**).

**Effectiveness outcomes**

*Renal function in untreated patients*

Annualized changes in eGFR slope (standard error) for untreated males (*n =* 112) and females (*n =* 454) aged ≥18 years were −.78 (.38) and −.82 (.11) mL/min/1.73 m^2^, respectively. This was similar for patients aged ≥16 years (male, −.78 [.38] mL/min/1.73 m^2^, *n =* 114; female, −.80 [.11] mL/min/1.73 m^2^, *n =* 462).

*Morbidity in untreated patients*

In untreated males and females, median (minimum, maximum) age at first composite event was 43.5 (.8, 86.9) years and 41.5 (2.3, 94.7) years, respectively. Similar data were seen when calculated using means (standard deviation): 42.8 (18.7) years and 43.0 (16.8) years for males and females, respectively. The estimated (95% confidence interval) age at which 50% of the untreated group had experienced a first composite event was 64.7 (59.4–67.5) years for males and 71.3 (69.1–74.6) years for females.

*Mortality in untreated patients*

For untreated males and females, median (minimum, maximum) ages at death were 68.5 (37.9, 83.6) years and 76.6 (40.0, 85.2) years, respectively. Similar data were seen when calculated as means (standard deviation): 65.7 (11.5) years and 71.8 (13.7) years for males and females, respectively. The estimated ages at which 50% of untreated males and females died were not calculable owing to high survival rates. The estimated (95% confidence interval) probability of surviving at least 10 years, 15 years and 19 years was .897 (.824–.941), .897 (.824–.941) and not calculable, respectively, in untreated male patients, and .984 (.970–.992), .979 (.959–.989), and .979 (.959–.989), respectively, in untreated female patients.

**Safety outcomes with at-home agalsidase alfa treatment**

There were 2645 treatment-emergent adverse events (TEAEs) in patients who received at least one dose of agalsidase alfa at home (**Table S10**). Of these TEAEs, 34.4% were mild and 31.7% were moderate by severity. Most reported TEAEs were determined not to be related to agalsidase alfa treatment by the investigator (91.0% [2406/2645]). In total, 1570 TEAEs (59.4%) were considered non-serious. Death was reported as a fatal SAE in 86 patients (10.1%) receiving agalsidase alfa at home. Of these, 83 (96.5%) were classified as not related to treatment; the relationship of the remaining three to treatment was not recorded.

**Supplementary Tables**

**Table S1.** Number of adults (treated and untreated) with Fabry disease at entry into FOS by country.

| Country | Male (*n =* 1614) | Female  (*n =* 2402) | Total (*N =* 4016) |
| --- | --- | --- | --- |
| Argentina | 31 (1.9) | 50 (2.1) | 81 (2.0) |
| Australia | 59 (3.7) | 86 (3.6) | 145 (3.6) |
| Austria | 25 (1.5) | 29 (1.2) | 54 (1.3) |
| Belgium | 10 (.6) | 12 (.5) | 22 (.5) |
| Brazil | 23 (1.4) | 31 (1.3) | 54 (1.3) |
| Canada | 113 (7.0) | 204 (8.5) | 317 (7.9) |
| Czech Republic | 55 (3.4) | 89 (3.7) | 144 (3.6) |
| Denmark | 2 (.1) | 2 (.1) | 4 (.1) |
| Finland | 28 (1.7) | 63 (2.6) | 91 (2.3) |
| France | 47 (2.9) | 70 (2.9) | 117 (2.9) |
| Germany | 253 (15.7) | 458 (19.1) | 711 (17.7) |
| Hungary | 4 (.2) | 8 (.3) | 12 (.3) |
| Israel | 1 (.1) | 2 (.1) | 3 (.1) |
| Italy | 90 (5.6) | 104 (4.3) | 194 (4.8) |
| Japan | 238 (14.7) | 176 (7.3) | 414 (10.3) |
| Netherlands | 44 (2.7) | 69 (2.9) | 113 (2.8) |
| Portugal | 24 (1.5) | 36 (1.5) | 60 (1.5) |
| Russia | 40 (2.5) | 36 (1.5) | 76 (1.9) |
| Slovenia | 12 (.7) | 24 (1.0) | 36 (.9) |
| South Korea | 5 (.3) | 3 (.1) | 8 (.2) |
| Spain | 67 (4.2) | 77 (3.2) | 144 (3.6) |
| Switzerland | 38 (2.4) | 55 (2.3) | 93 (2.3) |
| Taiwan | 177 (11.0) | 370 (15.4) | 547 (13.6) |
| United Kingdom | 228 (14.1) | 348 (14.5) | 576 (14.3) |

*Note:* Values are n (%). Adults were defined as patients aged ≥18 years at baseline.
Abbreviation: FOS, Fabry Outcome Survey.

**Table S2.** Exposure to agalsidase alfa for adult patients with Fabry disease enrolled in FOS.

|  | **Male**  **(*n =* 957)** | **Female**  **(*n =* 907)** | **Overall**  **(*n =* 1864)** |
| --- | --- | --- | --- |
| Median (min, max) dose, mg/kg | .20 (.0, 1.0) | .20 (.1, .6) | .20 (.0, 1.0) |
| *n* (*n* missing) | 909 (48) | 857 (50) | 1766 (98) |
| Range of doses, *n* (%) | | | |
| <.15 mg/kg | 13 (1.4) | 5 (.6) | 18 (1.0) |
| .15–<.25 mg/kg | 876 (96.4) | 840 (98.0) | 1716 (97.2) |
| .25–<.40 mg/kg | 13 (1.4) | 11 (1.3) | 24 (1.4) |
| >.40 mg/kg | 7 (.8) | 1 (.1) | 8 (.5) |
| *n* (*n* missing) | 909 (48) | 857 (50) | 1766 (98) |
| Median (min, max) treatment frequency, days | 14.0 (7.0, 15.0) | 14.0 (7.0, 15.0) | 14.0 (7.0, 15.0) |
| *n* (*n* missing) | 194 (763) | 251 (656) | 445 (1419) |
| Range of treatment frequency, *n* (%) | | | |
| 5–10 days | 5 (2.6) | 1 (.4) | 6 (1.3) |
| 11–18 days | 189 (97.4) | 250 (99.6) | 439 (98.7) |
| *n* (*n* missing) | 194 (763) | 251 (656) | 445 (1419) |

Abbreviation: FOS, Fabry Outcome Survey.

**Table S3.** Reasons for discontinuation from FOS.

| Primary reason for discontinuation, *n* (%) | Adults  (*n =* 1503) |
| --- | --- |
| Registry terminated by sponsor  Death  Lost to follow-up  Other^a^ | 822 (54.7)  127 (8.4)  61 (4.1)  493 (32.8) |

*Note:* Values are *n* (%).

Abbreviation: FOS, Fabry Outcome Survey.
^a^ ‘Other’ included patients who moved away, requested discontinuation, no longer met the inclusion criteria and who met the exclusion criterion (enrolment in blinded clinical trials).

**Table S4.** Fabry disease gene variant classification and use of ACE-Is or ARBs according to baseline urinary protein level subgroup stratified by sex in adults with Fabry disease treated with agalsidase alfa only in FOS.

|  | Baseline urinary protein level | | |  |
| --- | --- | --- | --- | --- |
|  | **<.3**  **(*n =* 391)** | **≥.3 to <1.0 (*n =* 144)** | **≥1.0 (*n =* 105)** | |
| Genotype |  |  |  | |
| Male, *n* | 42 | 30 | 21 | |
| Classic, *n* (%) | 34 (81.0) | 25 (83.3) | 20 (95.2) | |
| Non-classic (including N215S), *n* (%) | 8 (19.0) | 5 (16.7) | 1 (4.8) | |
| *n* (*n* missing) | 137 | 57 | 48 | |
| Female, *n* | 58 | 18 | 7 | |
| Classic, *n* (%) | 56 (96.6) | 17 (94.4) | 7 (100) | |
| Non-classic (including N215S), *n* (%) | 2 (3.4) | 1 (5.6) | 0 | |
| *n* (*n* missing) | 154 | 39 | 29 | |
| Use of ACE-I or ARB at any time |  |  |  | |
| Male, *n* | 179 | 87 | 69 | |
| ACE-I, *n* (%) | 46 (25.7) | 46 (52.9) | 39 (56.5) | |
| ARB, *n* (%) | 40 (22.3) | 40 (46.0) | 36 (52.2) | |
| ACE-I or ARB, n (%) | 72 (40.2) | 70 (80.5) | 57 (82.6) | |
| Female, *n* | 212 | 57 | 36 | |
| ACE-I, *n* (%) | 62 (29.2) | 31 (54.4) | 22 (61.1) | |
| ARB use, *n* (%) | 53 (25.0) | 17 (29.8) | 17 (47.2) | |
| ACE-I or ARB, *n* (%) | 93 (43.9) | 38 (66.7) | 29 (80.6) | |

*Note*: Adults were defined as patients aged ≥18 years at baseline.

Abbreviations: ACE-I, angiotensin-converting enzyme inhibitor; ARB, angiotensin receptor blocker; FOS, Fabry Outcome Survey.

**Table S5.** Time to and age at first composite morbidity event (renal, cardiac or stroke event, or death) in adults treated with agalsidase alfa only in FOS, stratified by classic and non-classic Fabry disease and by sex.

|  | **Classic** | | **Non-classic (including N215S)** | |
| --- | --- | --- | --- | --- |
|  | **Male  (*n =* 284)** | **Female**  **(*n =* 303)** | **Male (*n =* 112)** | **Female (*n =* 45)** |
| Event rate, *n* (%) | | | | |
| Patients with at least one event | 175 (61.6) | 167 (55.1) | 62 (55.4) | 20 (44.4) |
| Patients whose data were censored | 109 (38.4) | 136 (44.9) | 50 (44.6) | 25 (55.6) |
| Time to first event, months | | | | |
| Median (95% CI) | 65.1 (46.3–87.5) | 66.3 (41.5–96.5) | 62.3 (33.6–96.0) | 95.5 (25.1–NC) |
| 25^th^ percentile (95% CI) | 9.3 (5.2–16.0) | 7.6 (5.0–12.0) | 21.0 (6.5–28.7) | 11.6 (1.5–27.1) |
| 75^th^ percentile (95% CI) | NC (172.0–NC) | NC (189.9–NC) | 191.7 (104.6–NC) | NC (NC–NC) |
| Age at first composite event, years | | | | |
| Median (min, max) | 36.8 (4.5, 71.5) | 51.2 (10.6, 83.5) | 61.8 (11.3, 77.3) | 61.3 (16.1, 85.0) |
| Age of 50% surviving composite event, estimate (95% CI), years | 42.9 (40.0–44.7) | 58.3 (56.9–60.2) | 65.3 (62.9–67.5) | 64.8 (62.3–73.3) |

*Note*: Adults were defined as patients aged ≥18 years at baseline.
Abbreviations: CI, confidence interval; FOS, Fabry Outcome Survey; max, maximum; min, minimum; NC, not calculable; SD, standard deviation.**Table S6.** Survival data for adults treated with agalsidase alfa only in FOS, stratified by classic and non-classic Fabry disease and by sex.

|  | **Classic** | | **Non-classic (including N215S)** | |
| --- | --- | --- | --- | --- |
|  | **Male  (*n =* 284)** | **Female**  **(*n =* 303)** | **Male (*n =* 112)** | **Female (*n =* 45)** |
| Survival, *n* (%) | | | | |
| Number of surviving patients | 256 (90.1) | 290 (95.7) | 100 (89.3) | 44 (97.8) |
| Number of patients who have died | 28 (9.9) | 13 (4.3) | 12 (10.7) | 1 (2.2) |
| Age at death, years | | | | |
| *n* | 28 | 13 | 12 | 1 |
| Median (min, max) | 59.1 (40.9, 69.6) | 66.6 (51.6, 87.7) | 70.8 (63.4, 79.6) | 69.8 (69.8, 69.8) |
| Age of 50% surviving death, estimate  (95% CI), years | 63.5 (62.7–69.6) | 87.7 (78.9–NC) | 77.7 (77.3–NC) | NC (NC–NC) |
| Probability of survival from baseline, estimate (95% CI) | | | | |
| Survival for 10 years | .953 (.915–.975) | .959 (.918–.979) | .874 (.766–.935) | .960 (.748–.994) |
| Survival for 15 years | .912 (.858–.946) | .926 (.861–.961) | .736 (.518–.867) | .960 (.748–.994) |
| Survival for 19 years | .859 (.785–.909) | .864 (.675–.947) | .736 (.518–.867) | .960 (.748–.994) |

*Note*: Adults were defined as patients aged ≥18 years at baseline.

Abbreviations: CI, confidence interval; FOS, Fabry Outcome Survey; max, maximum; min, minimum; NC, not calculable.

**Table S7.** Summary of TEAEs by preferred term occurring in at least 2% of adults with Fabry disease treated with agalsidase alfa only in FOS.

| **TEAE, n (%) [m] [%]** | **Male**  **(*n =* 957)** | **Female**  **(*n =* 907)** | **Total (*N =* 1864)** |
| --- | --- | --- | --- |
| Diarrhoea | 60 (6.3) [118] [2.5] | 23 (2.5) [27] [1.0] | 83 (4.5) [145] [2.0] |
| Headache | 37 (3.9) [51] [1.1] | 46 (5.1) [62] [2.4] | 83 (4.5) [113] [1.5] |
| Atrial fibrillation | 40 (4.2) [59] [1.2] | 41 (4.5) [54] [2.0] | 81 (4.3) [113] [1.5] |
| Arthralgia | 37 (3.9) [46] [1.0] | 33 (3.6) [42] [1.6] | 70 (3.8) [88] [1.2] |
| Fatigue | 35 (3.7) [43] [.9] | 33 (3.6) [41] [1.6] | 68 (3.6) [84] [1.1] |
| Pain in extremity | 38 (4.0) [47] [1.0] | 28 (3.1) [40] [1.5] | 66 (3.5) [87] [1.2] |
| Cardiac failure | 35 (3.7) [43] [.9] | 30 (3.3) [33] [1.3] | 65 (3.5) [76] [1.0] |
| Transient ischaemic attack | 35 (3.7) [43] [.9] | 29 (3.2) [39] [1.5] | 64 (3.4) [82] [1.1] |
| Dyspnoea | 30 (3.1) [49] [1.0] | 33 (3.6) [36] [1.4] | 63 (3.4) [85] [1.2] |
| Cerebrovascular accident | 30 (3.1) [38] [.8] | 32 (3.5) [47] [1.8] | 62 (3.3) [85] [1.2] |
| Oedema peripheral | 40 (4.2) [47] [1.0] | 22 (2.4) [24] [.9] | 62 (3.3) [71] [1.0] |
| Vertigo | 32 (3.3) [33] [.7] | 29 (3.2) [32] [1.2] | 61 (3.3) [65] [.9] |
| Depression | 30 (3.1) [32] [.7] | 27 (3.0) [33] [1.3] | 57 (3.1) [65] [.9] |
| Pneumonia | 37 (3.9) [47] [1.0] | 19 (2.1) [21] [.8] | 56 (3.0) [68] [.9] |
| Angina pectoris | 30 (3.1) [34] [.7] | 25 (2.8) [29] [1.1] | 55 (3.0) [63] [.9] |
| Nasopharyngitis | 39 (4.1) [76] [1.6] | 16 (1.8) [26] [1.0] | 55 (3.0) [102] [1.4] |
| Pyrexia | 43 (4.5) [73] [1.5] | 11 (1.2) [13] [.5] | 54 (2.9) [86] [1.2] |
| Nausea | 31 (3.2) [38] [.8] | 21 (2.3) [30] [1.1] | 52 (2.8) [68] [.9] |
| Renal failure | 42 (4.4) [49] [1.0] | 10 (1.1) [12] [.5] | 52 (2.8) [61] [.8] |
| Dizziness | 30 (3.1) [36] [.8] | 21 (2.3) [25] [.9] | 51 (2.7) [61] [.8] |
| Palpitations | 23 (2.4) [24] [.5] | 25 (2.8) [28] [1.1] | 48 (2.6) [52] [.7] |
| Infusion-related reaction | 28 (2.9) [52] [1.1] | 19 (2.1) [21] [.8] | 47 (2.5) [73] [1.0] |
| Abdominal pain | 25 (2.6) [40] [.8] | 20 (2.2) [22] [.8] | 45 (2.4) [62] [.8] |
| Myocardial infarction | 27 (2.8) [32] [.7] | 16 (1.8) [16] [.6] | 43 (2.3) [48] [.7] |
| Back pain | 25 (2.6) [32] [.7] | 17 (1.9) [18] [.7] | 42 (2.3) [50] [.7] |
| Tinnitus | 20 (2.1) [21] [.4] | 21 (2.3) [24] [.9] | 41 (2.2) [45] [.6] |
| Paraesthesia | 22 (2.3) [26] [.5] | 18 (2.0) [21] [.8] | 40 (2.1) [47] [.6] |
| Vomiting | 32 (3.3) [45] [.9] | 8 (.9) [8] [.3] | 40 (2.1) [53] [.7] |

*Note*: Adults were defined as patients aged ≥18 years at the time of the cut-off date. Percentage of patients is based on the total number of adult-treated patients. Percentage of adverse events is based on the total number of events experienced. Adverse events were coded using MedDRA version 24.0. Patients were counted only once for each preferred term.

Abbreviations: (%), percentage of patients; [%], percentage of adverse events; FOS, Fabry Outcome Survey; n, number of patients; [m], number of adverse events; TEAE, treatment-emergent adverse event.

**Table S8.** Summary of treatment-emergent SAEs (excluding fatal events) occurring in more than two adults with Fabry disease treated with agalsidase alfa in FOS, stratified by System Organ Class.

| **System Organ Class, n (%) [m] [%]** | **Male**  **(*n =* 957)** | **Female**  **(*n =* 907)** | **Total (*N =* 1864)** |
| --- | --- | --- | --- |
| Cardiac disorders | 147 (15.4) [273] [5.8] | 100 (11.0) [177] [6.7] | 247 (13.3) [450] [6.1] |
| Nervous system disorders | 101 (10.6) [176] [3.7] | 89 (9.8) [146] [5.5] | 190 (10.2) [322] [4.4] |
| Infections and infestations | 113 (11.8) [233] [4.9] | 44 (4.9) [63] [2.4] | 157 (8.4) [296] [4.0] |
| Renal and urinary disorders | 93 (9.7) [123] [2.6] | 17 (1.9) [21] [.8] | 110 (5.9) [144] [2.0] |
| Gastrointestinal disorders | 62 (6.5) [102] [2.2] | 41 (4.5) [59] [2.2] | 103 (5.5) [161] [2.2] |
| Injury, poisoning and procedural complications | 59 (6.2) [95] [2.0] | 35 (3.9) [44] [1.7] | 94 (5.0) [139] [1.9] |
| General disorders and administration-site conditions | 43 (4.5) [53] [1.1] | 28 (3.1) [35] [1.3] | 71 (3.8) [88] [1.2] |
| Respiratory, thoracic and mediastinal disorders | 30 (3.1) [40] [.8] | 30 (3.3) [37] [1.4] | 60 (3.2) [77] [1.0] |
| Musculoskeletal and connective tissue disorders | 34 (3.6) [47] [1.0] | 24 (2.6) [39] [1.5] | 58 (3.1) [86] [1.2] |
| Neoplasms benign, malignant and unspecified (including cysts and polyps) | 21 (2.2) [30] [.6] | 19 (2.1) [25] [.9] | 40 (2.1) [55] [.7] |
| Vascular disorders | 23 (2.4) [31] [.7] | 16 (1.8) [24] [.9] | 39 (2.1) [55] [.7] |
| Psychiatric disorders | 15 (1.6) [19] [.4] | 10 (1.1) [21] [.8] | 25 (1.3) [40] [.5] |
| Metabolism and nutrition  disorders | 14 (1.5) [16] [.3] | 9 (1.0) [14] [.5] | 23 (1.2) [30] [.4] |
| Ear and labyrinth disorders | 15 (1.6) [20] [.4)] | 8 (.9) [9] [.3] | 23 (1.2) [29] [.4] |
| Investigations | 15 (1.6) [16] [.3] | 8 (.9) [9] [.3] | 23 (1.2) [25] [.3] |
| Reproductive system and breast  disorders | 10 (1.0) [10] [.2] | 11 (1.2) [14] [.5] | 21 (1.1) [24] [.3] |
| Hepatobiliary disorders | 10 (1.0) [11] [.2] | 8 (.9) [10] [.4] | 18 (1.0) [21] [.3] |
| Pregnancy, puerperium and perinatal conditions | 0 (0) [0] [0] | 14 (1.5) [17] [.6] | 14 (.8) [17] [.2] |
| Skin and subcutaneous tissue disorders | 12 (1.3) [13] [.3] | 2 (.2) [2] [.1] | 14 (.8) [15] [.2] |
| Blood and lymphatic system disorders | 8 (.8) [10] [.2] | 5 (.6) [6] [.2] | 13 (.7) [16] [.2] |
| Surgical and medical procedures | 8 (.8) [8] [.2] | 5 (.6) [5] [.2] | 13 (.7) [13] [.2] |
| Immune system disorders | 8 (.8) [12] [.3] | 3 (.3) [3] [.1] | 11 (.6) [15] [.2] |
| Eye disorders | 5 (.5) [6] [.1] | 6 (.7) [7] [.3] | 11 (.6) [13] [.2] |
| Congenital, familial and genetic disorders | 7 (.7) [9] [.2] | 2 (.2) [2] [.1] | 9 (.5) [11] [.1] |
| Product issues | 4 (.4) [6] [.1] | 3 (.3) [3] [.1] | 7 (.4) [9] [1] |
| Endocrine disorders | 2 (.2) [2] [<.1] | 3 [.3] 3 [.1] | 5 (.3) [5] [.1] |

*Note*: Adults were defined as patients aged ≥18 years at the time of the cut-off date. Percentage of patients is based on the total number of adult-treated patients. Percentage of events is based on the total number of events experienced. Adverse events were coded using MedDRA version 24.0. Patients were counted only once within each system organ class and preferred term.

Abbreviations: (%), percentage of patients; [%], percentage of adverse events; FOS, Fabry Outcome Survey; n, number of patients; [m], number of adverse events.

**Table S9.** Clinical events occurring in Fabry disease patients after agalsidase alfa treatment (treated) or FOS enrolment (untreated).

| Clinical event^a^ | Treated  (*n =* 1864) | Untreated  (*n =* 1613) | Total (*N =* 3477) |
| --- | --- | --- | --- |
| Dialysis^b^ |  |  |  |
| Haemodialysis |  |  |  |
| *n* | 1139 | 1424 | 2563 |
| Yes, *n* (%) | 5 (.4) | 0 (.0) | 5 (.2) |
| Peritoneal dialysis |  |  |  |
| *n* | 1174 | 1441 | 2615 |
| Yes, *n* (%) | 2 (.2) | 0 (.0) | 2 (.1) |
| Unspecified dialysis |  |  |  |
| *n* | 1131 | 1443 | 2574 |
| Yes, *n* (%) | 3 (.3) | 0 (.0) | 3 (.1) |
| Kidney transplant |  |  |  |
| *n* | 1139 | 1434 | 2573 |
| Yes, *n* (%) | 2 (.2) | 0 (.0) | 2 (.1) |

^a^ Events were treatment emergent but not necessarily related to treatment.

^b^ The same patient may have received dialysis via different methods.

**Table S10.** Summary of TEAEs occurring on or after first treatment with agalsidase alfa at home in adults with Fabry disease.

| **TEAE category, n (%) [m] [%]** | **Male**  **(*n =* 397)** | **Female**  **(*n =* 458)** | **Total (*N =* 855)** |
| --- | --- | --- | --- |
| **TEAE** | 224 (56.4) [1565] [100] | 234 (51.1) [1080] [100] | 458 (53.6) [2645] [100] |
| TEAE by severity |  |  |  |
| Not recorded | 7 (1.8) [197] [12.6] | 26 (5.7) [249] [23.1] | 33 (3.9) [446] [16.9] |
| Mild | 39 (9.8) [565] [36.1] | 41 (9.0) [346] [32.0] | 80 (9.4) [911] [34.4] |
| Moderate | 58 (14.6) [514] [32.8] | 84 (18.3) [324] [30.0] | 142 (16.6) [838] [31.7] |
| Severe | 65 (16.4) [213] [13.6] | 52 (11.4) [127] [11.8] | 117 (13.7) [340] [12.9] |
| Fatal | 55 (13.9) [76] [4.9] | 31 (6.8) [34] [3.1] | 86 (10.1) [110] [4.2] |
| TEAE by relationship to treatment | | | |
| Not recorded | 2 (.5) [28] [1.8] | 6 (1.3) [43] [4.0] | 8 (.9) [71] [2.7] |
| Not related | 190 (47.9) [1438] [91.9] | 201 (43.9) [968] [89.6] | 391 (45.7) [2406] [91.0] |
| Possibly related | 16 (4.0) [55] [3.5] | 17 (3.7) [57] [5.3] | 33 (3.9) [112] [4.2] |
| Probably related | 16 (4.0) [44] [2.8] | 10 (2.2) [12] [1.1] | 26 (3.0) [56] [2.1] |
| TEAE by seriousness | | | |
| Not recorded | 0 (.0) [12] [.8] | 3 (.7) [22] [.2] | 3 (.4) [34] [1.3] |
| Serious | 171 (43.1) [625] [39.9] | 158 (34.5) [416] [38.5] | 329 (38.5) [1041] [39.4] |
| Non-serious | 53 (13.4) [928] [59.3] | 73 (15.9) [642] [59.4] | 126 (14.7) [1570] [59.4] |
| Treatment-emergent IRRs | 27 (6.8) [87] [5.6] | 23 (5.0) [44] [4.1] | 50 (5.8) [131] [5.0] |
| Treatment-emergent deaths | 55 (13.9) | 31 (6.8) | 86 (10.1) |
| Treatment-emergent deaths by relationship to treatment | | | |
| Not recorded | 2 (.5) | 1 (.2) | 3 (.4) |
| Not related | 53 (13.4) | 30 (6.6) | 83 (9.7) |

*Note*: Adults were defined as patients aged ≥18 years at the time of the cut-off date. Percentage of patients is based on the total number of adult-treated patients. Percentage of adverse events is based on the total number of events experienced. Events that could not be classified as an IRR (‘Yes’ or ‘No’) owing to lack of information were imputed as ‘No’.

Abbreviations: (%), percentage of patients; [%], percentage of adverse events; IRR, infusion-related reaction; n, number of patients; [m], number of adverse events; TEAE, treatment-emergent adverse event.

|  | **Male**  **(*n =* 957)** | **Female**  **(*n =* 907)** | **Total (*N =* 1864)** |
| --- | --- | --- | --- |
| Number receiving home therapy, *n* (%) | 397 (41.5) | 458 (50.5) | 855 (45.9) |
| Age at first home therapy, median (min, max), years | 42.6 (18.4, 76.7) | 51.0 (18.4, 80.8) | 46.9 (18.4, 80.8) |
| *n* (*n* missing) | 397 (560) | 458 (449) | 855 (1009) |
| Time from agalsidase alfa start to first treatment at home, median (min, max) years | 2.0 (0, 19.5) | 1.1 (0, 19.3) | 1.5 (0, 19.5) |
| Person administering home therapy, *n* (%) | | | |
| Nurse | 340 (85.6) | 392 (85.6) | 732 (85.6) |
| Relative | 24 (6.0) | 14 (3.1) | 38 (4.4) |
| Self-administration | 58 (14.6) | 60 (13.1) | 118 (13.8) |

**Table S11**. Clinical characteristics of adults with Fabry disease treated with agalsidase alfa at home at least once.

*Note*: Except for the age at first home therapy, there were no missing data for patients receiving home therapy.

Abbreviations: max, maximum; min, minimum.

**Table S12.** Summary of treatment-emergent IRRs in adults with Fabry disease treated with agalsidase alfa in FOS.

| **TEAE category, n (%) [m] [%]** | **Male**  **(*n =* 957)** | **Female**  **(*n =* 907)** | **Total (*N =* 1864)** |
| --- | --- | --- | --- |
| Treatment-emergent IRR | 153 (16.0) [778] [100] | 88 (9.7) [229] [100] | 241 (12.9) [1007] [100] |
| Treatment-emergent IRR by severity | | | |
| Not recorded | 63 (6.6) [504] [64.8] | 36 (4.0) [107] [46.7] | 99 (5.3) [611] [60.7] |
| Mild | 46 (4.8) [163] [21.0] | 31 (3.4) [86] [37.6] | 77 (4.1) [249] [24.7] |
| Moderate | 35 (3.7) [97] [12.5] | 18 (2.0) [28] [12.2] | 53 (2.8) [125] [12.4] |
| Severe | 8 (.8) [13] [1.7] | 3 (.3) [8] [3.5] | 11 (.6) [21] [2.1] |
| Fatal^a^ | 1 (.1) [1] [.1] | 0 (.0) [0] [.0] | 1 (.1) [1] [.1] |
| Treatment-emergent IRR by relationship | | | |
| Not recorded | 2 (.2) [2] [.3] | 3 (.3) [3] [1.3] | 5 (.3) [5] [.5] |
| Possibly related | 73 (7.6) [258] [33.2] | 46 (5.1) [122] [53.3] | 119 (6.4) [380] [37.7] |
| Probably related | 78 (8.2) [518] [66.6] | 39 (4.3) [104] [45.4] | 117 (6.3) [622] [61.8] |
| Treatment-emergent IRR by seriousness | | | |
| Serious | 18 (1.9) [28] [3.6] | 16 (1.8) [25] [10.9] | 34 (1.8) [53] [5.3] |
| Non-serious | 135 (14.1) [750] [96.4] | 72 (7.9) [204] [89.1] | 207 (11.1) [954] [94.7] |
| Treatment-emergent IRR deaths | 1 (.1) | 0 (.0) | 1 (.1) |
| Treatment-emergent IRR deaths by relationship | | | |
| Possibly related^b^ | 1 (.1) | 0 (.0) | 1 (.1) |

*Note*: Adults were defined as patients aged ≥18 years at the time of the cut-off date. Percentage of patients is based on the total number of adult-treated patients. Percentage of events is based on the total number of events experienced. Events that could not be classified as an IRR (‘Yes’ or ‘No’) due to lack of information were imputed as ‘No’.

Abbreviations: (%), percentage of patients; [%], percentage of adverse events; FOS, Fabry Outcome Survey; IRR, infusion-related reaction; n, number of patients; [m], number of adverse events; TEAE, treatment-emergent adverse event.

^a^ Data for number of fatal TEAEs differ from those reported in the *mortality* section because not all were reported as TEAEs in the database.

^b^ One IRR of acute cardiac failure, possibly related to treatment, had a fatal outcome.

**Table S13**. Summary of hospitalizations during the study period.

| Event | Treated  (*n =* 1864) | Untreated  (*n =* 1613) | Total (*N =* 3477) |
| --- | --- | --- | --- |
| Hospitalizations, *n* (%) | 595 (31.9) | 255 (15.8) | 850 (24.4) |
| Number of hospitalizations, mean (SD) | 2.6 (2.6) | 1.6 (1.2) | 2.3 (2.3) |
| Length of hospital stay, median (Q1, Q3) days | 7.0 (3.0, 17.0) | 5.0 (3.0, 18.0) | 6.0 (3.0, 17.0) |
| Reason for hospital admission |  |  |  |
| Surgery, *n* (%) | 274 (17.6) | 64 (15.6) | 338 (17.2) |
| Cardiovascular disease, *n* (%) | 196 (12.6) | 38 (9.3) | 234 (11.9) |
| Infection, *n* (%) | 122 (7.8) | 14 (3.4) | 136 (6.9) |
| Fabry disease evaluation, *n* (%) | 85 (5.5) | 30 (7.3) | 115 (5.9) |
| Stroke/cerebrovascular accident, *n* (%) | 69 (4.4) | 18 (4.4) | 87 (4.4) |
| Gastrointestinal, *n* (%) | 58 (3.7) | 14 (3.4) | 72 (3.7) |
| Renal disease, *n* (%) | 49 (3.2) | 13 (3.2) | 62 (3.2) |
| Pulmonary, *n* (%) | 40 (2.6) | 11 (2.7) | 51 (2.6) |
| Orthopaedic, *n* (%) | 38 (2.4) | 12 (2.9) | 50 (2.5) |
| Neurologic, *n* (%) | 27 (1.7) | 9 (2.2) | 36 (1.8) |
| Psychiatric, *n* (%) | 25 (1.6) | 8 (2.0) | 33 (1.7) |
| Malignancy/chemotherapy, *n* (%) | 18 (1.2) | 10 (2.4) | 28 (1.4) |
| Accident, *n* (%) | 23 (1.5) | 5 (1.2) | 28 (1.4) |
| Other, *n* (%) | 475 (30.5) | 149 (36.3) | 624 (31.8) |
| *n* (*n* missing) | 1555 (2) | 410 (0) | 1965 (2) |

*Note:* Reasons for hospitalizations reported in >1.5% of patients in any group are presented.
Abbreviations: max, maximum; min minimum; Q, quartile; SD, standard deviation.

**References**

1. Elstein DAGBM. *Fabry disease.* Dordrecht: Springer; 2010.

2. Eng CM, Ashley GA, Burgert TS, Enriquez AL, D'Souza M, Desnick RJ. Fabry disease: thirty-five mutations in the alpha-galactosidase A gene in patients with classic and variant phenotypes. *Mol Med.* 1997;3(3):174-182.

3. Germain DP, Oliveira JP, Bichet DG, et al. Use of a rare disease registry for establishing phenotypic classification of previously unassigned GLA variants: a consensus classification system by a multispecialty Fabry disease genotype-phenotype workgroup. *J Med Genet.* 2020;57(8):542-551.

4. Lee TH, Yang JT, Lee JD, et al. Genomic screening of Fabry disease in young stroke patients: the Taiwan experience and a review of the literature. *Eur J Neurol.* 2019;26(3):553-555.

5. Lukas J, Giese AK, Markoff A, et al. Functional characterisation of alpha-galactosidase a mutations as a basis for a new classification system in fabry disease. *PLoS Genet.* 2013;9(8):e1003632.

6. Malavera A, Cadilhac DA, Thijs V, et al. Screening for Fabry disease in young strokes in the Australian Stroke Clinical Registry (AuSCR). *Front Neurol.* 2020;11:596420.

7. Martins AM, Cabrera G, Molt F, et al. The clinical profiles of female patients with Fabry disease in Latin America: A Fabry Registry analysis of natural history data from 169 patients based on enzyme replacement therapy status. *JIMD Rep.* 2019;49(1):107-117.

8. Nampoothiri S, Yesodharan D, Bhattacherjee A, et al. Fabry disease in India: A multicenter study of the clinical and mutation spectrum in 54 patients. *JIMD Rep.* 2020;56(1):82-94.

9. Ortiz A, Germain DP, Desnick RJ, et al. Fabry disease revisited: Management and treatment recommendations for adult patients. *Mol Genet Metab.* 2018;123(4):416-427.

10. Parini R, Pintos-Morell G, Hennermann JB, et al. Analysis of renal and cardiac outcomes in male participants in the Fabry Outcome Survey starting agalsidase alfa enzyme replacement therapy before and after 18 years of age. *Drug Des Devel Ther.* 2020;14:2149-2158.

11. Pavlu L, Kocourkova L, Taborsky M, Petrkova J. Ventricular tachycardia: a presentation of Fabry disease case report. *Eur Heart J Case Rep.* 2019;3(1):yty154.

12. Rekova P, Dostalova G, Kemlink D, et al. Detailed phenotype of GLA variants identified by the nationwide neurological screening of stroke patients in the Czech Republic. *J Clin Med.* 2021;10(16).

13. Sawada T, Kido J, Sugawara K, et al. Detection of novel Fabry disease-associated pathogenic variants in Japanese patients by newborn and high-risk screening. *Mol Genet Genomic Med.* 2020;8(11):e1502.

14. Sezer O, Ceylaner S. Genetic management algorithm in high-risk Fabry disease cases; especially in female indexes with mutations. *Endocr Metab Immune Disord Drug Targets.* 2021;21(2):324-337.

15. Shabbeer J, Yasuda M, Benson SD, Desnick RJ. Fabry disease: identification of 50 novel alpha-galactosidase A mutations causing the classic phenotype and three-dimensional structural analysis of 29 missense mutations. *Hum Genomics.* 2006;2(5):297-309.

16. Thomas DC, Sharma S, Puri RD, Verma IC, Verma J. Lysosomal storage disorders: Novel and frequent pathogenic variants in a large cohort of Indian patients of Pompe, Fabry, Gaucher and Hurler disease. *Clin Biochem.* 2021;89:14-37.

17. Saito S, Ohno K, Sakuraba H. Fabry-database.org: database of the clinical phenotypes, genotypes and mutant alpha-galactosidase A structures in Fabry disease. *J Hum Genet.* 2011;56(6):467-468.

18. Desnick RJ, Chen R, Srinivasan R, Doheny DO, Bishop D. The Fabry disease genotype-phenotype database (dbFGP): an international expert consortium. *Molecular Genetics and Metabolism.* 2017;120(1-2):S41-S42.

19. Oder D, Liu D, Hu K, et al. alpha-Galactosidase A genotype N215S induces a specific cardiac variant of Fabry disease. *Circ Cardiovasc Genet.* 2017;10(5).

20. Koulousios K, Stylianou K, Pateinakis P, et al. Fabry disease due to D313Y and novel GLA mutations. *BMJ Open.* 2017;7(10):e017098.
